# High Prevalence of Metabolic Syndrome among Kuwaiti Adults —A Wake-Up Call for Public Health Intervention

**DOI:** 10.3390/ijerph9051984

**Published:** 2012-05-23

**Authors:** Sameer Al Zenki, Husam Al Omirah, Suad Al Hooti, Nawal Al Hamad, Robert T. Jackson, Aravinda Rao, Nasser Al Jahmah, Ina'am Al Obaid, Jameela Al Ghanim, Mona Al Somaie, Sahar Zaghloul, Amani Al Othman

**Affiliations:** 1 Kuwait Institute for Scientific Research, PO Box 24885, Safat 13109, Kuwait; Email: omirah@kisr.edu.kw (H.A.O.); shooti@kisr.edu.kw (S.A.H.); jsager@kisr.edu.kw (J.A.G.); aothman@kisr.edu.kw (A.A.O.); 2 Ministry of Health, Food and Nutrition Administration, PO Box 24225, Safat 13103, Kuwait; Email: nutrition90@hotmail.com (N.A.H.); alsomaie@hotmail.com (M.A.S.); 3 Department of Nutrition and Food Science, University of Maryland, College Park, MD 20742, USA; Email: bojack53@gmail.com; 4 Ministry of Health, Medical Laboratories Services, Sabah Hospital Laboratories, PO Box 24225, Safat 13103, Kuwait; Email: aravindarao@hotmail.com (A.R.); nasseraljahmah@yahoo.com (N.A.J.); inaob218@hotmail.com (I.A.O.); 5 National Nutrition Institute, 16 Kasr El Aini Street, Cairo, Egypt; Email: szaghloul@kisr.edu.kw

**Keywords:** metabolic syndrome, obesity, gender differences, international definitions, epidemiology, cardiovascular diseases, Kuwait

## Abstract

The socio-economic development which followed the discovery of oil resources brought about considerable changes in the food habits and lifestyle of the Kuwaiti population. Excessive caloric intake and decreased energy expenditure due to a sedentary lifestyle have led to a rapid increase in obesity, diabetes and other non-communicable chronic diseases in the population. In this paper, we examine the prevalence of the Metabolic Syndrome (MetS) among Kuwaiti adults (≥20 years) using data from the first national nutrition survey conducted between July 2008 and November 2009. The prevalence of MetS was 37.7% in females and 34.2% in males by NCEP criteria, whereas the values were 40.1% in females and 41.7% in males according to IDF criteria. Prevalence of MetS increased with age and was higher in females than males. The high prevalence of the MetS in Kuwaiti adults warrants urgent public health measures to prevent morbidity and mortality due to cardiovascular complications in the future.

## 1. Background

Kuwait is small country with a total population of 2,595,628 (July 2011 estimate), 45% of whom are Kuwaiti citizens [1]. Citizens enjoy a high standard of living that includes free universal education and medical care. The discovery of oil in the 1950s brought about dramatic changes in living standards and food availability in the country [2,3,4,5]. Because of high *per capita* income (estimated to be $48,900 in 2010) and food subsidies provided by the government, there is a wide access to food for all Kuwaiti citizens. An increased caloric intake with a concomitant decrease in levels of physical activity are thought to be the main causes of high rates of overweight and obesity [1,6,7,8,9,10,11]. It has been estimated that about one-third of Kuwaiti males and one-half of females are obese [6,12,13,14]. 

Worldwide, obesity has been shown to pose a major risk for a variety of debilitating and life-threatening chronic conditions, including cardiovascular disease, Type 2 Diabetes Mellitus (T2DM), hypertension and stroke, gall bladder disease, osteoarthritis and certain cancers [15]. Already in Kuwait Type 2 Diabetes Mellitus is reported to be a growing public health concern [13,14,15,16,17]. 

Individuals with overall and/or abdominal obesity have an increased risk of developing metabolic disorders such as metabolic syndrome (MetS). MetS is characterized by abdominal obesity, increased blood pressure, triglycerides levels, fasting blood glucose levels, and by lower HDL cholesterol levels [15,18]. Individuals who possess three or more (of the five) abnormal components or indicators have been shown to have a two-fold increased risk of cardiovascular disease and a five-fold increased risk for T2DM [18,19].

Studies from around the world, the Arabian Gulf and Kuwait in particular, where the nutrition transition has been particularly rapid and dramatic [1] show that obesity is increasing both in prevalence and also severity [6]. Individuals with obesity have an increased risk of having components of MetS [6]. However, despite the plethora of studies from other areas of the world, there is a paucity of data on MetS in Kuwait. What has been learned about MetS in Kuwait is largely from small hospital-based studies, from other patient-based studies, or from studies performed in a single geographical area of the country.

The purpose of this study was to examine the prevalence of MetS and its components among Kuwaiti adults who were representative of the general population in order to understand its distribution in males and females. We also sought to understand how MetS prevalence differed by age and body mass index in this population. This is the first study of MetS prevalence that uses a nationally representative sample of adult Kuwaitis and that also compares MetS prevalence using two of the most widely used definitions of the MetS, *i.e.*, the USA National Cholesterol Education Program Adult Treatment Panel III (NCEP ATPIII) and the International Diabetes Federation (IDF) definitions. 

## 2. Methods

### 2.1. Sample Selection

The protocols for the national nutrition survey were approved by the Ethics Committee of the Ministry of Health and thereafter the data were collected from seven primary health care centers of the Ministry between July 2008 and November 2009. An informed consent was also obtained from each participant. A multistage stratified cluster sample of 1,830 Kuwaitis was selected based on 2005 Census data. Subjects were sampled from 545 separate Kuwaiti households from all six geographical areas or governorates (strata). Governorates were divided into localities proportionate to Kuwaiti population density and then into clusters. From each cluster 20 households were selected using stratified sampling. At the household level, subjects were randomly selected from each age category taking into consideration census gender distribution. The current study represents data on adult Kuwaitis ≥20 years of age. Socioeconomic, health, anthropometry and blood indices were obtained. 

### 2.2. Laboratory Procedures

Fasting blood samples were collected with minimal stasis by qualified phlebotomists and were then transported to and processed at the Sabah Hospital National Reference Laboratory. Blood glucose, triglycerides, HDL-cholesterol, and total cholesterol were analyzed on a Dade-Behring (Siemens) Dimension RxL automated clinical chemistry analyzer, using the manufacturer’s kits and protocols. Harmonized cut-off points [18] for the MetS risk components were used. The following harmonized abnormal components are the same for both the NCEP ATPIII and the IDF definitions of MetS: fasting plasma glucose ≥ 5.6 mmol/L; HDL cholesterol-women < 1.30 mmol/L; men < 1.04 mmol/L; triglyceride ≥ 1.70 mmol/L (150 mg/dL); Systolic BP ≥ 130 mmHg or Diastolic BP ≥ 85 mmHg. The WC cut-off for NCEP is 102 cm for men and 88 cm for women; while the IDF cut-off point is ≥94 cm for men and ≥80 cm for women.

### 2.3. Anthrpometry

Height was measured using a Seca 214 stadiometer (Hanover, MD, USA) to the nearest 0.1 cm. Weight and body fat percentage were measured using the TANITA model TBF 310 body composition analyzer (Arlington Heights, IL, USA). Body mass index was calculated and categorized based on WHO standards [20] (overweight as BMI ≥ 25 kg/ m^2^ and obesity as BMI ≥ 30 kg /m^2^). Body fat percentage (BF%), body fat weight (BF), fat free mass (FFM) and total body water (TBW) were also recorded from the analyzer. Waist circumference was measured at minimal respiration midway between the iliac crest and lower rib area. 

### 2.4. Measurement of Blood Pressure

Blood pressure was measured with a sphygmomanometer after subjects had been seated for at least five minutes. 

### 2.5. Assessment of Physical Activity

Activity levels of subjects were self-reported as “sedentary”, “lightly active”, “moderately active”, or “very active”.

### 2.6. Data Analysis

Data were analyzed using the SAS (version 9.2, SAS Institute, Cary, NC, USA). Due to the complex sampling design, appropriate weights were used in the data analysis. Descriptive statistics (means and frequencies) were calculated. Non-normally distributed variables (e.g., triglycerides and glucose) were log transformed. Logistic regression was used to calculate the odds of having abnormal components with increased age (compared to 20–49 years), sex (males compared to females) and body mass index (BMI levels in the overweight and obese ranges compared to normal). A significant result was taken to be *P* < 0.05.

## 3. Results

Males and non-pregnant females comprised 44.7% and 55.3% of the sample, respectively and were drawn from all regions of the country. Table 1 shows several background characteristics of Kuwaiti adult (≥20 years) males and females. The mean ages of males and females were 39.1 ± 0.9 and 40.9 ± 0.7, respectively and ranged between 20 and 86 years. For individuals with less than high school, a similar percentage of females (42.6%) had elementary school or less education compared to males (44%). Similar percentages of males and females reported that they had completed high school and college. 

**Table 1:** Background characteristics of Kuwaiti adults by gender.

Characteristics	Men (459)	Women (533)	*P*
	Mean SEM	Mean SEM	
**Age (years)**	39.1 0.9	40.9 0.7	0.101
**Waist circumference (cm)**	99.5 1.3	92.2 1.2	<0.001
**BMI (kg/m²)**	28.5 0.6	30.9 0.5	<0.001
**% FAT**	23.3 0.7	37.7 0.7	<0.001
**Fat Free Mass**	63.0 0.7	46.0 0.4	<0.001
**Blood Pressure (mmHg)**			
**Systolic**	131.3 1.1	126.4 1.2	<0.001
**Diastolic**	83.2 0.8	80.7 0.7	<0.001
**Glucose (mmol/L) ^‡^**	5.9 0.1	5.9 0.1	0.658
**HBA1C (%) ^‡^**	6.1 0.1	6.0 0.1	0.552
**Lipid Profile (mmol/L)**			
**Total cholesterol**	4.9 0.1	5.0 0.1	0.448
**Triglycerides ^‡^**	1.2 0.0	1.0 0.0	0.271
**HDL–cholesterol**	1.1 0.0	1.3 0.0	<0.001
**Education (%)**			
**<high school**	42.6 3.5	44.0 3.4	0.773
**High school**	35.9 2.8	36.7 2.9	
**College and above**	21.4 2.5	19.3 2.3	
**Activity level (%)**			
**Lightly active**	58.3 3.1	68.4 2.8	<0.001
**Moderately active**	24.0 2.8	16.3 1.9	
**Sedentary**	12.9 2.1	13.8 1.8	
**Very active**	4.7 1.2	1.3 0.6	

Data expressed as mean ± standard error and percentage for categorical data; **^‡^** geometric means-variables log transformed prior to analysis.

The majority of the sample reported their activity levels as being “lightly active”. Small percentages of males (4.7% ± 1.2%) and females (1.3 ± 0.6%) reported their activity levels as being “very active”. 

**Figure 1:**
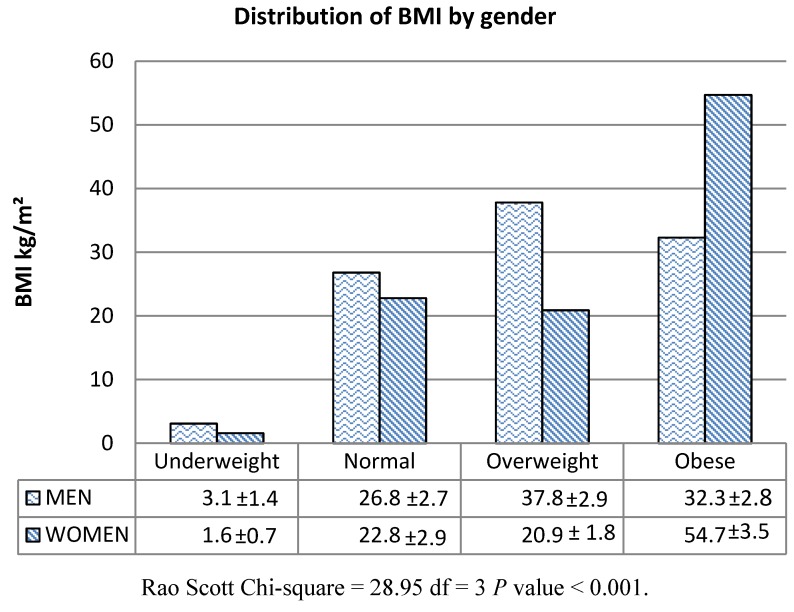
Distribution of BMI by gender in Kuwaiti adults.

Waist circumferences (WC) of males were significantly greater (*P* < 0.001) than those of females; however, the BMI and % body fat levels of females were significantly greater (*P* < 0.001) than those for males. A higher percentage of males were overweight than were females (37.8% *vs.* 20.9%); however a significantly greater (*P* < 0.001) percentage of females were obese compared to males (32.3% *vs.* 54.7%) (Figure 1).

Males had higher (*P* < 0.001) mean systolic and diastolic blood pressures than did females. However the mean blood glucose levels and the HbA1c levels of Kuwaiti males and females were similar (*P* > 0.05).

Mean levels of total blood cholesterol and triglyceride did not differ significantly between males and females. However, females had significantly higher (*P* < 0.001) HDL cholesterol values than males (1.3 ± 0.02 *vs.* 1.1 ± 0.02 mmol/L).

**Figure 2:**
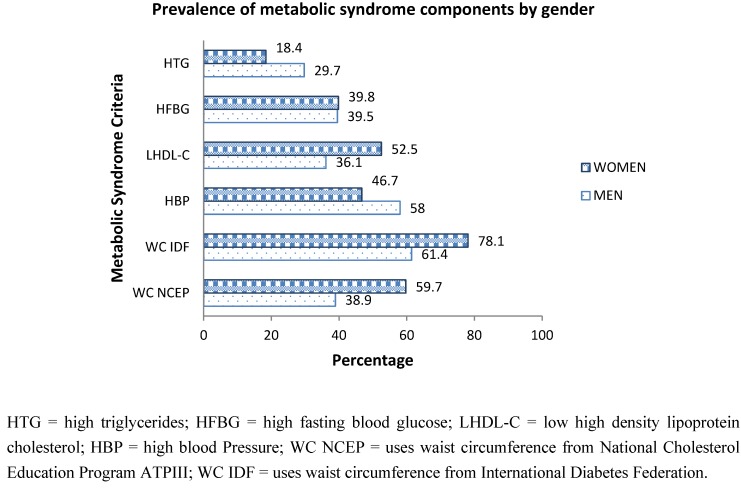
Overall prevalence of the metabolic syndrome components by gender in Kuwaiti adults.

Figure 2 shows the prevalence of MetS components in adults by gender. Males had a higher prevalence of abnormal levels of serum triglyceride and also high blood pressure compared to adult females. Females had a higher prevalence of abnormal levels of fasting plasma glucose and low HDL cholesterol levels compared to adult males. Abdominal obesity was the most prevalent MetS abnormality for both genders followed by high blood pressure for men and low HDL levels in women.

Since the cut-off points for WC differ between the NCEP and IDF definitions, we estimated the percentage of males and females that would be adjudged to have abnormally high WC according to each definition. Figure 2 shows that by both definitions the prevalence of abnormally high WC values is greater in females than in males. However, the magnitude of the estimates of abnormal WC varied considerably. Thus, the prevalence of abnormal WC in females is 59.7% by NCEP and 78.1% by IDF. In males the NCEP cut point gave 38.9% and the IDF gave a prevalence estimate of 61.4% with abnormal WC values.

**Figure 3:**
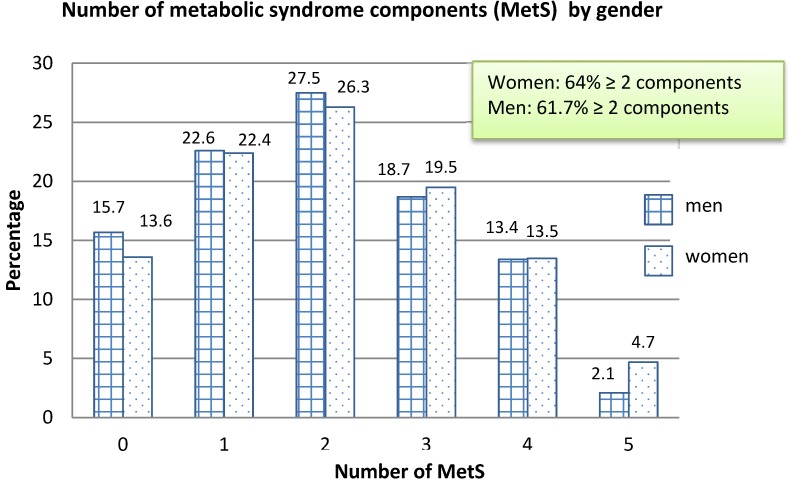
Number of metabolic syndrome components by gender in Kuwaiti adults.

Figure 3 shows the number of MetS components present in Kuwaiti adults by gender by the NCEP criteria. Thus the MetS was more prevalent in females (37.7%) than in males (34.2%). By the IDF definition 41.7% of males and 40.1% of females had MetS (Table 2). A slightly higher percentage of males (15.7%) than females (13.6%) had no MetS component abnormalities. Males also had higher percentages of one (22.6% *vs.* 22.4%) and two (27.5% *vs.* 26.3%) MetS abnormalities compared to females. Females had greater percentages of three, four, and five abnormal metabolic components than males did. More than twice the percentage of females had all five metabolic abnormalities indicative of the MetS. Overall, 64% of females and 61.7% of males had two or more abnormal MetS indicators.

**Table 2:** Unadjusted and age-specific prevalence of metabolic syndrome in Kuwaiti adults.

	N		MetS ** NCEP Syndrome		MetS ***IDF Syndrome
			Mean SEM		Mean SEM
**Unadjusted Age**	992		36.1 1.9		40.9 2.0
**Men**	459		34.2 2.8		41.7 3.1
**Women**	533		37.7 2.7		40.1 2.8
**Men**					
**20–40**	192		23.4 3.9		29.5 4.6
**40–60**	181		46.9 5.1		53.8 4.7
**60+**	86		50.1 6.1		66.4 6.2
**Women**					
**20–40**	207		17.3 3.1		18.2 3.1
**40–60**	251		51.1 4.0		54.5 3.9
**60+**	75		65.4 6.6		70.8 6.2

** MetS-Metabolic Syndrome defined using WC ≥ 102 cm in men and ≥ 88 cm in women (NCEP/ATP). *** MetS-Metabolic Syndrome defined using WC ≥ 94 cm in men and ≥ 80 cm in women (IDF).

Table 2 compares the prevalence of the MetS by age group using the NCEP and IDF definitions for MetS. The IDF definition for the MetS gives consistently higher prevalence rates than does the NCEP definition. By each definition the prevalence of the MetS is seen to increase with increased age group (in both genders). Overall, without adjusting by age groupings, 37.7% of women and 34.2% of men meet the NCEP criteria for the MetS, compared to 40.1% of women and 41.7% of men who meet the IDF criteria for MetS. 

The prevalence of the MetS is higher among males at younger ages, e.g., 20–30 years (18.5%) compared to females (9.7%). 50–60 year old males have the highest prevalence of the MetS. The opposite was observed in females, where the oldest age group (≥60 years) had the highest prevalence of MetS (having three or more abnormal components). Those who were in the age category 40–59 years (Table 3) were 2.5 (95% Confidence interval (CI: 1.6–3.9)] times as likely as those in the 20–39 year age category to meet the criteria for having MetS. However, those in the ≥60 year age category were 4.9 times (95% CI: 2.7–8.8) as likely to meet the MetS criteria. 

Subjects with a BMI in the “overweight” range were more than 4.8 times (95% CI: 2.4–9.6) as likely as those with a BMI in the “normal’ range to meet the criteria for the MetS; while those in the “obese” groups were 19.7 times more likely to have MetS (95% CI: 9.7–40.2) (Table 3). 

**Table 3:** Odds of Having MetS by Age, Gender and BMI status among Kuwaiti Adults Odds ratios for prevalence of metabolic syndrome among adults ≥ 20 years of age.

Characteristics	Odds ratio (95% confidence interval)	
**Age**		
20–39 years	1.00	NA
40–59 years	2.47 *	1.57–3.88
60 years and over	4.89 *	2.73–8.77
**Gender**		
Females	1.0	NA
Males	1.58 **	1.05–2.38
**Body mass index (kg/m²)**		
normal weight	1.00	NA
Overweight	4.77 *	2.36–9.63
Obese	19.69 *	9.65–40.18

NA: category not applicable. Reference groups: age group 20-39 years; female; normal BMI. * *p* value < 0.001; ** *p* value < 0.05.

**Figure 4:**
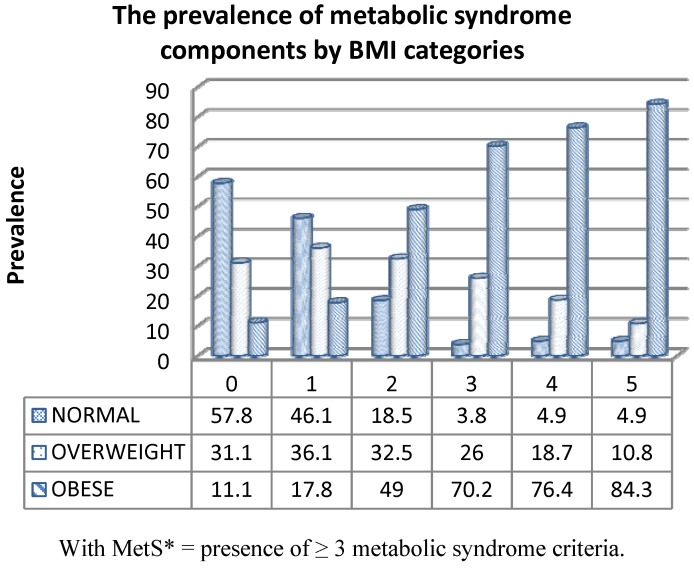
Prevalence of metabolic syndrome* components by BMI categories in Kuwaiti adults.

Figure 4 shows that as BMI categories increase (from normal to overweight to obese) the percentage of subjects who have higher numbers of abnormal MetS components also increased. For example, in subjects with “normal” weight only 3.8% and 4.9%, respectively, have three or four abnormal MetS indicators. In subjects in the “overweight” category, 25.9% and 18.7% of subjects have three or four abnormal metabolic indicators. In the “obese” category, however, 70.2% and 76.4% of the subjects, respectively, have three or four abnormal metabolic indicators.

## 4. Discussion

MetS represents a significant risk for the development of cardiovascular diseases and T2DM [12,15]. Yet, in Kuwait like many other less developed countries, there is a paucity of data available on the prevalence of this problem to alert clinicians, for public health intervention, or for international comparisons. Moreover, for most non-European populations there is a lack of consensus as to which definition of MetS is most appropriate for use [15]. 

This study represents the first nationally representative study of MetS undertaken in Kuwait that estimates the overall prevalence of MetS, the prevalence of MetS components and compares the prevalence of MetS using the two most commonly used definitions of MetS (NCEP ATP III and IDF). We also examine MetS prevalence by gender, by BMI level, and by age in this first Kuwaiti National Nutrition Survey.

The prevalence of MetS among Kuwaiti adults differed according to the definition that was utilized (Table 2). According to the NCEP ATP III criteria the prevalence of MetS was 37.7% in females and 34.2% in males. However according to the IDF definition, the prevalence of MetS was 40.1% in females and 41.7% in males. By either definition, our estimated prevalence is somewhat higher than that found previously in Kuwaitis and also among other Arab populations studied. For example, Al Shaibani *et al.* [21] studying a sample of 609 adult males and females in one governorate of Kuwait found, using the ATPIII guidelines, that MetS was prevalent in 32.8% of their subjects. In 2005, Al Sultan *et al.* [22] found (using the IDF definition) that 33.9% of Kuwaiti diabetic patients met the criteria for MetS. Al Rashdan and Al Nesef [23] estimated that 36.2% of Kuwaitis met the criteria for MetS (by the IDF definition). Our current estimate using the same IDF definition, but in a non-diabetic nationally representative sample of Kuwaitis found that slightly over 40% of Kuwaitis meet the criteria for MetS. Perhaps this increased prevalence of MetS may be an indication of a temporal increase, since from all accounts obesity is increasing in Kuwait [1,3,6,7,8] and obesity, as measured by BMI or WC, is the major determinant of the differences in the estimates of the most common definitions of MetS. 

The IDF definition, compared with the NCEP ATP III definition, showed a higher prevalence of MetS among Saudi Arabian adults and likewise our study showed that obesity was a predominant contributor to MetS prevalence among males and females [24]. In another Gulf Arab study, Al Lawati *et al.* [25] found using the NCEP ATP III definition that the age-adjusted prevalence of MetS was 19.5% among men and 23.0% among adult Omani females. Thus our results from Kuwait show the highest prevalence of MetS found so far among the Arabian Gulf countries.

Also of concern is that the prevalence of MetS in Kuwait is higher than that reported from some developed countries, such as the USA, where the prevalence of MetS among adults 20 years and above was recently reported to be 34% [26]. In another USA study, in a random sample of 542 men and non-pregnant Arab American women aged 20–75 years the prevalence of MetS was found to be 23% (95% CI: 19–26%) by the ATP III definition and 28% (CI: 24–32%) by the WHO definition [27]. Although the breakdown of the Arab ethnic groups in that sample was not specified, few were likely to be Gulf Arabs, since their numbers are low in the USA population.

As found previously in other populations [26,27,28], the prevalence of MetS among Kuwaitis differed by gender (Table 2). Kuwaiti females have a higher overall prevalence than males [11,29,30]. In some western populations, on the other hand, the prevalence of MetS was higher in males than in females [28]. 

MetS prevalence and risk also differed by age (Table 2 and Table 3). For example in the 20–40 year age group males had a higher prevalence of MetS, however in the 40–60 year age group and the 60 and above year age groups, women had higher prevalence of MetS. Although using the NCEP ATP III and IDF criteria for MetS gave different prevalence estimates, the patterns of MetS prevalence by age was the same by each definition (Table 2) with a general tendency for MetS to increase with increasing age. Other investigators have also reported that MetS prevalence increases [23,26,27] as age increased. 

The mean BMI and body fat percentage of females was greater than that for males (Table 1). Three-quarters (75.6%) of Kuwaiti females and 70.1% of males were overweight (BMI ≥ 25.0 kg/m^2^) or obese (BMI ≥ 30.0 kg/m^2^). Obesity (both overall and abdominal) has been found to be a significant public health problem in Kuwait previously [4,6,31] and seems to be increasing [3,9] among all segments of the Kuwaiti population, especially among females, where 55% are obese [3,9,10,11] and the problem seems to start in childhood and adolescence [8].

The body fat percentage of females was greater than that for males. Carr *et al.* [32] showed that as body fat increases, so do the numbers of abnormal metabolic indicators individuals are likely to possess. Our data show this also (Figure 4), for as BMI increases across categories (from normal to overweight to obese) the percentage of subjects who have higher numbers of abnormal MetS components also increase. In Kuwaitis in the “normal” weight category, only 3.8% and 4.9% respectively, have three or four abnormal MetS components. However in the “obese” category 70.2% and 76.4% of the subjects, respectively, had either three or four abnormal MetS components. 

The overwhelming majority of males (71.2%) and females (82.2%) classified themselves as “sedentary” or “lightly active. The high rates of “inactivity” or “low activity” coupled with high rates of overweight and obesity pose a significant current and future risk for cardiovascular diseases in this population [5,19]. Some studies [33,34,35] already show that T2DM is higher in Kuwait than in most other countries of the World [22]. In a recent study [36] of 484 young adults of both sexes between 17 and 24 years of age, for example, the prevalence of impaired glucose regulation (impaired fasting glucose, impaired glucose tolerance, and elevated HbA1c levels was 32%. In a systematic review of overweight, obesity, hyperglycemia, hypertension and dyslipidemia in the Gulf, Al Hyas *et al.* [16], found a high prevalence of risk factors for diabetes and diabetic complications in the Gulf Cooperation Council region, especially in Kuwait.

## 5. Conclusions

In conclusion, the prevalence of MetS differed significantly depending on which of the common definitions is used (NCEP ATP III or IDF). The major difference between these definitions is the cut-points used for waist circumference. Currently, Kuwait has not yet developed accepted waist circumference or BMI cut-points for adults [31]. 

When these are developed a more precise, ethnically specific estimate of the MetS for Kuwaitis and similar Gulf Arab populations will be possible. Usage of the extant definitions demonstrates that the prevalence of MetS is alarmingly high. Although Kuwaiti, or a Gulf specific anthropometric cut-off value for WC and BMI do not yet exist and their absence poses a major limitation in estimating MetS prevalence in this study, clinicians and public health authorities should take immediate action to reduce abnormal MetS components among Kuwaitis. These actions should center on lowering caloric intake and increasing caloric expenditure and explaining through public health messages the nexus between obesity and cardiovascular diseases.
